# Stigma, lost autonomy, reclaimed identity, and uncertainty around safe movement: a qualitative interview study of physical activity in gout

**DOI:** 10.1007/s00296-026-06265-3

**Published:** 2026-07-28

**Authors:** Sarah Stewart, Libby Anderson, Tania Ka’ai, Karl Rudolph, Julie Collis, Nicola Kayes, David Rice, Irene Zeng, Nicola Dalbeth

**Affiliations:** 1 School of Allied Health, Faculty of Environmental Sciences, Auckland University of Technology, Auckland, New Zealand; 2 Department of Medicine, Faculty of Medical and Health Sciences, University of Auckland, Auckland, New Zealand; 3 Te Ipukarea Research Institute, Auckland University of Technology, Auckland, New Zealand; 4 Department of Allied Health, School of Health Sciences, Swinburne University, Melbourne, Australia; 5 Centre for Person Centred Research, Auckland University of Technology, Auckland, New Zealand; 6 Pain Service, Health New Zealand Te Whatu Ora Waitematā, Auckland, New Zealand; 7 Clinical Sciences Management, Faculty of Health and Environmental Sciences, Auckland University of Technology, Auckland, New Zealand; 8 Health New Zealand, Te Whatu Ora Te Toka Tumai, Auckland, New Zealand

**Keywords:** Arthritis, Gouty, Exercise, Motor Activity, Qualitative Research, Interviews as Topic

## Abstract

To explore patients’ experiences and perceptions of physical activity in the context of gout and gout flares. Interpretive description informed the design of this qualitative study. Individual interviews were conducted with 25 people with gout. An interview guide was used to lead discussions, focusing on participants’ experiences of gout flares, physical activity engagement, and the perceived impact of flares on activity levels. Key questions were designed to capture culturally specific understandings and experiences related to gout and physical activity. Interviews were audio-recorded and transcribed verbatim. Data were analysed using reflexive thematic analysis. Four themes were generated from the data: (1) The experience of physical activity is shaped by societal misconceptions about gout; (2) A loss of physicality comes with a loss of autonomy; (3) Reclaiming body and identity through physical activity; and (4) Living with uncertainty: wanting to be active but not knowing how. Gout flares disrupted mobility, independence, work, family roles, and identity. Participants described stigma, fear of judgement, activity modification, pacing and uncertainty about whether exercise could trigger flares or damage joints, particularly in the absence of gout-specific advice. This study offers new insights into how societal misconceptions about gout influence the patient experience of physical activity, including loss of autonomy, identity reconstruction through movement, and uncertainty around safe exercise. These findings may inform future intervention development, and patient-centred physical activity guidance that supports people with gout to move safely, confidently, and meaningfully.

**Supplementary Information:**

The online version contains supplementary material available at 10.1007/s00296-026-06265-3.

## Introduction

Gout is a chronic condition characterised by recurrent flares of intense pain, swelling, and reduced joint mobility [1]. Gout flares can significantly impact function, recreational activities and activities of daily living [2–4]. People with gout experience extreme difficulty or complete inability to walk during gout flares, and can also have major upper limb impairment, including difficulties with hand use [2–4]. Physical exertion or joint injury has also been reported by some as a trigger for flares [5], and many people with gout avoid exercise and sports activities that could involve bumping or injuring a joint for fear of provoking a flare [4, 6, 7].

Current research also shows that people with gout have significantly lower levels of physical activity compared to people without gout [8]. This poses an important and under-recognised challenge, as “regular exercise” has been recommended in gout management guidelines to improve overall health and prevent or manage common comorbidities such as cardiovascular disease, obesity, metabolic syndrome, diabetes, and hypertension [9]. However, published management guidelines lack specific recommendations regarding exercise frequency, intensity, or safe approaches to physical activity for people with gout [9]. Recent literature has further highlighted physical activity, including walking, as a potentially important but underdeveloped area in gout prevention and management [10].

No research has explicitly explored patients’ perceptions and lived experiences of physical activity in the context of gout and gout flares. Understanding these perspectives is essential for developing strategies and gout-specific exercise recommendations that support safe, regular physical activity.

The aim of this study was to explore patients’ experiences and perceptions of physical activity in the context of gout and gout flares.

## Methods

### Study design

This qualitative study followed the Standards for Reporting Qualitative Research [11] (**Supplementary File 1**) and employed interpretive description [12, 13], an applied health research methodology that generates practical knowledge to inform health care delivery [14].

### Study rigour

The multidisciplinary team included expertise in podiatry, occupational therapy, physiotherapy, health psychology, and rheumatology. In Aotearoa New Zealand, gout is highly prevalent among Māori (Indigenous People of Aotearoa) and Pacific Peoples due to a genetic predisposition to high urate levels [15]. These populations also experience disproportionately early onset of gout, with frequent flares and major physical impairment [16–18]. Cultural rigour was supported by a Māori cultural advisor and academic (TK, Ngāti Porou and Ngāi Tahu) and a Māori patient research partner (KR, Ngā Puhi and Tai Poutini) with 30 years of lived experience with gout. Two experienced qualitative researchers (JC, NK) supported analytical depth. The first author (SS), a Pākehā (New Zealand European) registered podiatrist and academic with 15 years of gout research experience, led data collection and analysis. Reflexive practices were embedded throughout, and all authors reviewed and refined the themes.

### Participants

Adults aged *≥* 20 years were eligible if they had a self-reported physician diagnosis of gout and met the 2015 ACR/EULAR gout classification criteria [19]. The ACR/EULAR criteria were reviewed with each participant during eligibility screening, including clinical features, serum urate where available, and relevant clinical history. Microscopy confirmation or imaging evidence was not required for inclusion. Participants were recruited via social media, local health clinics, and existing research databases. Purposive sampling was used to obtain diversity in ethnicity, gout disease duration, and flare history. Guided by information power [20], a sample size of 25 participants was set to enable in-depth exploration. Information power was supported by the focused study aim, the specificity of participants lived experiences of gout and gout flares, the richness and relevance of the interview data, and the analysis of patterns across participants to generate clinically relevant themes [20]. Sampling was therefore directed towards generating depth and variation relevant to the research aim rather than achieving statistical representativeness or data saturation. Ethical approval for the study was obtained from the Auckland University of Technology (AUT) Ethics Committee (AUTEC reference: 23/305; approval date: 26/10/2023). All participants provided informed consent prior to participation. Participants interviewed in person provided written consent, while participants interviewed via Zoom or telephone had the option of providing recorded oral consent, as per the approved ethics protocol.

### Data collection

Between January 2024 and February 2025 individual interviews were conducted in-person, online via Zoom, or over the phone, based on participant preference. Interviews were led by SS and/or LA (Ngāti Maniopoto-Waikato) using a semi-structured interview guide (**Supplementary File 2**). The interview guide was used flexibly, with prompts and probes to explore responses in depth. The interview guide was developed through the research team’s clinical, methodological, and cultural expertise, and was informed by previous qualitative interview studies exploring the patient experience of gout and gout flares, literature on physical activity in gout, and current gout management guidelines [2–4, 6–10]. Questions explored experiences of gout flares, physical activity, perceptions of activity as a flare trigger, and culturally specific understandings relevant to gout and activity. Reflexive journaling and iterative refinement ensured responsiveness to participants’ lived experiences. Physical activity was defined as “*any bodily movement produced by skeletal muscles that results in energy expenditure*” [21], including exercise, and everyday activities such as household chores, stair use and transportation. Demographic and medical data were also recorded. Participants completed the International Physical Activity Questionnaire (IPAQ) short version [22] which categorised physical activity as ‘low’, ‘moderate’ or ‘high’. All interviews were audio-recorded and transcribed verbatim by SS and LA. Interviews were conducted in English and no formal language translation was required. Culturally responsive interviewing was supported through the involvement of Māori team members and advisors, and the interview guide was used flexibly to allow participants to describe culturally specific understandings in their own terms. All participants had the opportunity to review their transcripts prior to analysis, and two participants did so, although no substantive changes were requested.

### Data analysis

Data were analysed inductively using the six-phase process of reflexive thematic analysis [23, 24]. The lead researcher (SS) maintained reflexive journal entries throughout to document evolving interpretations and analytic decisions [25]. Interview recordings and transcripts were reviewed multiple times to allow familiarisation. Initial coding was completed using the comment function in Microsoft Word and involved the generation of distinct labels for pieces of information that were relevant to the research aim. A selection of codes was sense-checked by the qualitative advisors (JC, NK) to encourage multiple interpretations of the data [24]. To enhance credibility, an iterative approach was taken with the coding, which involved codes being reviewed and refined to capture deeper, latent meanings [26]. The final codes were transferred to a Microsoft Excel spreadsheet along with their associated data segments using a thematic analysis coding macro [27]. The codes were then organised visually on miro.com (an online collaborative platform). During theme development, codes were grouped within and across participants and progressively merged into broader clusters where shared meanings, recurring patterns, or conceptual overlap were identified. These clusters were developed into candidate themes, which were then refined through in-depth team discussions focused on what each theme encompassed, how themes related to one another, and whether they captured meaningful patterns across the coded data and full transcripts. Candidate themes were revised, combined, renamed, or set aside if they were too descriptive, overlapping, or insufficiently connected to the study aims. Reviewing and refining of themes involved collaborative discussion with the research team to ensure each theme functioned well as meaningful and distinct interpretations of the data.

## Results

### Participant characteristics

Twenty-five participants with gout were included (Table 1**)**. Participants were aged 32–67 years, and all but one were male. Ethnicities were Māori (24%), Pacific Peoples (24%), Asian (8%), and European (44%). Gout duration ranged from 2 to 30 years. Most participants reported recurrent flares and just over half used long-term urate-lowering therapy. Interviews were conducted in-person (*n* = 14, 56%), online over Zoom (*n* = 5, 20%), or over the phone (*n* = 6, 24%). Most participants lived in Auckland, New Zealand (*n* = 18, 72%). The mean (range) length of the interviews was 41.0 (23–59) minutes.

**Table 1:** Participant demographic and clinical characteristics (*n* = 25)

Age, years	Mean (SD)	47.9 (11.3)
	Range	32–67
Gender, n (%)	Female	1 (4%)
	Male	24 (96%)
Ethnicity	Māori	6 (24%)
	Pacific Peoples	6 (24%)
	Asian	2 (8%)
	European	11 (44%)
Gout disease duration, years	Mean (SD)	9.6 (7.2)
	Range	2–30
Number of flares in previous six months	Mean (SD)	2.8 (2.9)
	Range	0–10
Medication use, n (%)	Urate lowering therapy	15 (60%)
	Prednisone	8 (32%)
	Colchicine	7 (28%)
	NSAIDs	14 (56%)
	Other analgesic	4 (16%)
	Rongoā Rākau	1 (4%)
Comorbidities, n (%)	Diabetes	3 (12%)
	Cardiovascular disease	5 (20%)
	Kidney disease	2 (8%)
	Depression/anxiety	3 (12%)
IPAQ-SF, MET-minutes/week, mean (SD)	Walking	2284.3 (3551.9)
	Moderate	1016.8 (1777.3)
	Vigorous	2260.8 (3206.6)
	Total physical activity	5561.9 (4912.7)
IPAQ-SF, physical activity classification, n (%)	Low	6 (24%)
	Moderate	2 (8%)
	High	17 (68%)

IPAQ-SF International Physical Activity Questionnaire-Short Form

### Themes

Four themes were generated: (1) The experience of physical activity is shaped by societal misconceptions about gout; (2) A loss of physicality comes with a loss of autonomy; (3) Reclaiming body and identity through physical activity; and (4) Living with uncertainty: wanting to be active but not knowing how. A glossary of Māori terms is provided in Table 2.

**Table 2:** Glossary of te Reo Māori terms^a^

Mana	Prestige, authority, control, power, influence, status, spiritual power, charisma - mana is a supernatural force in a person, place or object.
*manaakitanga*	Hospitality, kindness, generosity, support - the process of showing respect, generosity and care for others.
*moko* (short for *mokopuna*)	Grandchild/grandchildren.
*tino rangatiratanga*	Self-determination, autonomy, self-government, control, power.
*whakamā*	To be ashamed, embarrassed, self-conscious.
*whānau*	An extended family or family group, traditionally the core economic unit in Māori society, now also used to include close friends with strong relational ties.

^*a*^Adapted from Te Aka Māori Dictionary [Mobile app]. Te Whanake. Retrieved 13 August 2025,* from Apple - App Store.*

#### Theme 1. The experience of physical activity is shaped by societal misconceptions about gout

The context in which participants experienced and navigated physical activity was governed by societal misconceptions around gout which were dominated by gout being an ‘old man’s disease’ associated with gluttony and overindulgence in food and alcohol. Due to this societal discourse, within which the experience of physical activity was situated, physical disability caused by gout was inherently connected to feelings of *whakamā* – a concept encompassing shame, embarrassment, and self-consciousness.

*Whakamā* was not only a response to physical limitations but also to the social judgement participants felt from others. The visibility of gout symptoms, such as the “gout limp,” became a symbol of their condition and a target for ridicule.

*“in like the older generations and my whānau* [family], *so I remember my uncle John you know um*,* would always have gout or one of the other uncles would always have gout and they’d be limping around and people would say oh you have to give up the booze you know and stuff like that”* (P003, Māori, F, gout duration: 8 years).

*”oh no*,* he hasn’t walked for a week. He’s limping around with big red foot ha*,* ha*,* ha*,* ha ha”* (P017, Māori, M, gout duration: 30 years).

Such reactions intensified participants’ *whakamā*, making them feel exposed and misunderstood. This was especially evident in situations where physical activity was expected, such as school camps or family outings.

*“And*,* you know*,* school camps is all about being outdoors and doing lots of activities and stuff. And yeah*,* I know-*,* like*,* partway through the camp*,* I got a flare. And I asked to be-*,* to not be a part of the activities that they were doing. Yeah. And I was a bit kind of like*,* at that point-*,* I was like*,* quite embarrassed*,* quite ashamed”* (P016, Asian, M, gout duration: 6 years).

*“go down the stairs very very you know one step at a time*,* I’m like an 80 year old*,* and I feel like that sometimes and you know even my grandkids say “hurry up Nan” you know but I have to take it really really slow and that gets really embarrassing*,* that’s embarrassing for me”* (P003, Māori, F, gout duration: 8 years).

People with gout felt that their reduced capacity for physical activity, particularly during gout flares, was underestimated and misunderstood by others, including friends, family, and employers. Participants felt that people who had not experienced gout themselves were unable to appreciate the extent of physical disability or empathise with people with gout.

*“you worry that people like-*,* like-*,* they don’t realise how much pain you’re in and so you kind of-*,* you kind of feel like a fake cause like*,* all they see is like a swollen joint and you know it’s a bit red or something*,* like they don’t*,* they don’t know how debilitating it can be” (*P002, European, M, gout duration: 2 years*)*.

#### Theme 2. A loss of physicality comes with a loss of autonomy

Gout flares caused intense pain and severe physical limitations, leaving participants unable to perform even basic tasks. This loss of physicality disrupted daily routines, led to a loss of autonomy, and for many, a diminished sense of *mana* (personal authority and dignity). People with gout described a loss of control over their bodies and felt physically dependent on others, which often affected their self-worth.

People with gout described being immobile and bedridden, particularly during the peak pain period of their flares.

*“Oh*,* this thing here*,* gout*,* when it comes over-*,* when I get a gout attack*,* it just shuts me down. Shuts me down of everything that I do”.* (P021, Pacific Peoples, M, gout duration: 6 years)

All day-to-day tasks requiring movement were impacted during a flare, including the ability to fulfil personal, work-related and *whānau* (family) roles.

*“Things are kept to a minimal*,* so toilet*,* unless I’m really*,* really busting*,* I’m not about to get up and go to the toilet*,* just because I want to urinate*,* so I really try my best to hold it. Showers*,* I try to minimise to like*,* if I don’t have to*,* I try once*,* and it’ll probably be like every third day*,* I really try my best to not go and do anything*,* eating is the same again*,* like unless I’m really starving or hungry*,* I really won’t get myself out of bed just to go and get something to eat or drink”* (P019, Māori, M, gout duration: 3 years).

Feelings of physical helplessness during flares were coupled with dependency on others. Participants described feeling they were a burden to others, which often led to feelings of frustration.

*“I don’t know if my kids have been scarred by what they’ve had to do … Particularly trying to get up*,* get clean… And then take my size*,* six foot four and a big man. You know*,* that’s a hard body to help. Particularly when the person’s-*,* you can’t touch the person and you’re yelling and screaming in pain. And you don’t know what to do and nothing’s working.”* (P017, Māori, M, gout duration: 30 years).

Although these physical limitations were often temporary, occurring only during flares, their unpredictability imposed a persistent mental burden. Participants described the constant uncertainty around when a flare might strike, which disrupted routines, forced cancellations, and made planning ahead difficult. This impacted their ability to confidently engage in meaningful physical activity.

*“I was still worried that if I went for a 2km walk or a 3km walk*,* I was concerned that halfway through the walk I’d get it again. And then I’d be struggling to get back to where I needed to be*,* back home or back to the car”* (P011, European, M, gout duration: 4 years).

#### Theme 3. Reclaiming control of body and identity through physical activity

Most people with gout recognised and were motivated by the value of physical activity which was regarded as a core component of overall health. Participants had a strong sense of *tino rangatiratanga* (self-determination) in how they conceptualised physical activity through various pacing and modification strategies. In this respect, physical activity was a means to reframe their identity to include, but not be defined, by their gout.

Physical activity was viewed as a valuable means to maintain a healthy weight, which for some participants was associated with a reduction in flare frequency and an improved ability to cope with the physical impact of gout flares. Physical activity was not only a way to manage gout, but also a means of maintaining mobility, ageing well, and managing comorbidities.

*“My dad sort of um passed on. He had a heart attack*,* I think*,* in his early 60s and stuff. So*,* you know*,* I just want to try and keep active. Also*,* I know that especially resistance training increases bone density and with mum falling and breaking bones recently over the last few years*,* it’s something that I’m pretty vigilant about myself too*,* just trying to keep active in that regard”.* (P020, Pacific Peoples, M, gout duration: 25 years)

Physical activity also supported mental wellbeing. Exercise helped with relaxation and re-energising, and helped people with gout take their mind off their condition.

*“I’m cleaning around everywhere and playing with the moko* [grandchildren], *mowing the lawns and I just put my mind off the gout you know*,* all that gout stuff”* (P001, Māori, M, gout duration: 12 years).

People with gout had a strong sense of self-awareness and respect for their bodies and physical abilities. Activity modification and pacing strategies were recognised as valuable methods to maintain activity.

*“I want to go from here to that tree which is about 25 yards and that’s it*,* and I’ll come back and have a sit down and I’ll go from there to there*,* and I won’t do the whole lot in one 10 minutes*,* I’ll do it over a period of 2–3 hours*,* you know*,* come back in here*,* make a sandwich*,* go back out*,* have a cup of tea and then finish it off”* (P001, Māori, M, gout duration: 12 years).

As part of reconceptualising physical activity in the context of living with gout, participants used ingenious and creative solutions to maintain movement during flares.

*“So most times I’ll just drop the knee onto the wheelie chair*,* my office chair*,* and then start wheeling myself around like that. And it’s okay if you’ve got um hard wood floors or tiles and stuff like that*,* but we’ve got carpet*,* so wood floors into carpet into wood floors again*,* so it’s not just smooth rolling*,* there’s a little bit more work involved*,* but not as much as getting around on the crutches”* (P019, Māori, M, gout duration: 3 years).

Importantly, participants described how regular use of urate-lowering therapy (ULT) helped them feel more in control of their gout and more confident in engaging with physical activity consistently and safely.

*“Now a few years on*,* I feel like if I continue to stay on my medication*,* and stay regular with that medication*,* by and large*,* exercise that I enjoy doing*,* like walking*,* is not going to trigger an attack.”* (P011, European, M, gout duration: 4 years).

Physical activity didn’t need to be strenuous to be meaningful and enjoyable. Participants found value in modified, everyday activities that aligned with their priorities and family life.

*“I think me just finding something that I enjoy*,* and me having reasons*,* and understanding the importance of life and time*,* I think that’s sort of been more of a-*,* yeah a reason for me to have been active … when you start seeing your children grow up*,* … and then you start seeing your grandchildren come through*,* you realise that something as simple as going to the park is quite big*,* on the bigger scale of schemes.”* (P019, Māori, M, gout duration: 3 years).

Many embraced a mindset of perseverance, refusing to let the physical disability caused by gout define their lives.

*“Oh*,* it’s frustrating*,* but I still get out a lot. I just mowed the lawns this morning. I still get out and do what I can*,* even though I’m walking funny. I don’t let it stop me completely. I don’t shut up shop and sit on the couch. I still work around it”.* (P008, Māori, M, gout duration: 8 years)

#### Theme 4. Living with uncertainty: Wanting to be active but not knowing how

Although all participants with gout expressed a strong desire to exercise, many felt uncertain about how to do so safely in the context of gout. Fears that exercise might trigger flares, worsen symptoms, or cause long-term joint damage led to anxiety and avoidance. The absence of clear, consistent advice from healthcare professionals further compounded these concerns and contributed to a deep sense of lacking *manaakitanga* - care and support.

Although people with gout valued the importance of physical activity for overall health, they questioned the value of exercise for gout-specific management. This uncertainty was driven by the experience of discomfort following exercise and the continuation of gout flares despite leading an active lifestyle.

*“I don’t know if exercising will help because when-*,* I was… After I got the gout attacks and I did some exercise*,* you know*,* when you’re running and doing some exercise*,* I still had the gout attacks. So I don’t know if exercising is another trigger. But it’s so unclear to me of what I could be doing”.* (P025, Pacific Peoples, M, gout duration: 8 years)

Many participants also expressed concern about the potential for physical activity to cause permanent joint damage, particularly when exercising during or shortly after a flare.

*“So my concern is I don’t want the crystals in my joint to be bugging up my joints when I get even older. Yeah. So that’s my major concern*,* is what damage am I doing to myself by exercising when I have gout?”* (P012, European, M, gout duration: 20 years).

Confusion around the role of movement in triggering flares or worsening symptoms created a persistent fear of exercise. This fear added to the mental load of managing gout and made it difficult for people to confidently enjoy physical activity.

*“Yeah*,* I mean*,* it just kind of*,* you know*,* it gave me one little extra thing to worry about. Which*,* you know*,* for a person who’s trying to put all their effort into making*,* you know*,* healthy decisions to do more exercise*,* like you don’t need one extra thing to worry about”.* (P016, Asian, M, gout duration: 6 years)

The lack of clear, consistent, and gout-specific guidance from healthcare professionals amplified this uncertainty and left participants feeling unsupported. People with gout wanted to be active, but needed guidance that acknowledged their lived experience, respected their concerns, and empowered them to move safely and confidently. Lifestyle advice was often limited to dietary changes, while advice on physical activity was either absent or conflicting.

*“The only advice I’ve probably gotten is I have to cut out things like*,* you know*,* shellfish from my diet. That’s the only thing*,* because I don’t smoke and I don’t drink. So*,* that could be the only thing. So*,* cut out shellfish*,* eat more greens kind of thing. But not basically*,* you know*,* do-*,* nothing about physical exercise”* (P025, Pacific Peoples, M, gout duration: 8 years).

*“We’re not told if you get straight back and do it* [physical activity], *is that good or bad? You know*,* where’s our plan? Where’s our management plan? Where’s my management plan? After 30 years*,* where’s my plan? Who do I ask for a plan? … Where is the*,* um*,* medical expertise and my guidance?”* (P017, Māori, M, gout duration: 30 years).

## Discussion

This study provides insights into how people with gout experience physical activity. Societal misconceptions shaped participants’ experiences of physical limitation, loss of autonomy, attempts to reclaim identity through movement, and uncertainty about how to safely exercise. Previous studies have highlighted barriers to physical activity and the need for tailored physical activity guidance in other inflammatory arthritis, including rheumatoid arthritis, which highlights patients’ desire for more comprehensive discussions with health professionals [28] and evidence that people with inflammatory arthritis experience greater barriers to physical activity [29]. The present study extends this physical activity literature to gout, showing that engagement in movement with gout is not only shaped by pain and flare-related disability, but also by social judgement, loss of autonomy, disruption to identity, uncertainty about movement safety, and the absence of gout-specific physical activity guidance.

The first theme highlighted how outdated views about gout influenced participants’ experiences of physical activity. Despite strong evidence that genetic predisposition plays a central role in gout development [30], public discourse continues to emphasise lifestyle blame, reinforcing unempathetic societal views [31–33]. A previous longitudinal study of people with gout found that negative illness perceptions predicted worsening functional status over time, independent of baseline disability [34]. Visible gout symptoms, such as limping or swelling, may therefore carry social and emotional consequences beyond the physical impairment itself. Promoting accurate and culturally responsive illness narratives may reduce stigma, drive behavioural change, and support positive engagement in physical activity.

Participants’ narratives also showed how gout flares cause temporary but profound disablement, not just through pain, but through dependence on others and loss of *mana* (personal authority and dignity) (Theme 2). This loss of independence is well-documented in gout literature [7, 35–39] and quantitative studies report high rates of work disability and productivity loss [40, 41]. Internalised expectations of independence and productivity may diminish self-worth when personal or whānau roles cannot be fulfilled. The loss of *mana*, central to Māori and Pacific worldviews, reflects a loss of personal dignity, authority, and meaningful contribution [42, 43].

While Theme 2 highlighted the loss of autonomy during flares, Theme 3 revealed how participants reclaimed agency through adaptive and meaningful engagement with activity. They showed resilience by modifying routines, pacing activities, and repurposing household items to enable activity; strategies consistent with self-management approaches in chronic musculoskeletal conditions [44]. This self-determination, or *tino rangatiratanga*, reflects an approach grounded in Māori wellbeing models that emphasise autonomy, cultural values, and meaningful contribution [45]. Participants emphasised being experts of their own bodies, knowing what they could tolerate during flares and how best to respond to physical limitations. This underscores the need for individualised activity plans that consider personal context, joint health, and flare timing. Simple, low-cost tools in familiar home environments can also reduce barriers to movement.

The final theme emphasised that motivation was not the barrier to exercise, but rather uncertainty about how to exercise safely and effectively with gout. This challenges assumptions that inactivity in gout stems from laziness and instead highlights a gap in knowledge and support. Clinically, these findings suggest that healthcare professionals should provide physical activity counselling as part of gout management. People with gout may benefit from clear, gout-specific guidance on safe movement during and after flares, pacing, joint protection, gradual return to activity, urate-lowering therapy, and personalised activity plans tailored to flare patterns, comorbidities, goals, and cultural contexts.

Lived experiences must inform future guidelines to ensure relevance, particularly for Māori and Pacific peoples who bear a disproportionate burden of gout in Aotearoa [16–18]. Experiences of pain-related restriction, uncertainty about safe movement, and limited physical activity advice were evident across participants. However, the meanings attached to these experiences were culturally situated. Some Māori and Pacific participants described physical limitation in relation to whānau roles, dignity, contribution, and expectations of strength and responsibility. This highlights the need for physical activity guidance that is not only clinically specific to gout, but also responsive to the social, cultural, and relational contexts in which people live, move, work, and care for others.

This study has strengths and limitations. Inclusion of a patient research partner and a cultural advisor ensured grounding in patient voice and culturally responsive practices. However, the small number of participants in some ethnic groups, particularly Asian participants, limited detailed comparison of culturally specific experiences. The study only included one female participant, which does not reflect the typical 3:1 male-to-female ratio of gout [46]. Women’s experiences of physical activity in the context of gout may differ from men’s, particularly in relation to caregiving responsibilities, social expectations, stigma, help-seeking, and the impact of mobility limitations on family, work, and daily roles. Future research should specifically explore the experiences of women with gout to better understand these potentially distinct perspectives. Although recruitment was undertaken through multiple routes, including social media, local health clinics, and existing gout research databases, participation may have been influenced by self-selection. The recruitment methods used may have favoured people who were more engaged with health care, health-conscious, or willing to discuss physical activity. Those involved also had higher self-reported engagement in physical activity, which may indicate that the sample included people who were more engaged with, positive about, or able to participate in physical activity. People who are more sedentary, have more severe disability or comorbidities may have been less able or willing to participate. This may have shaped the themes, particularly those relating to resilience, adaptation, and reclaiming identity through movement. Although medication use and flare frequency were recorded, differences in urate-lowering therapy use, adherence, disease control, and flare frequency were not explored in depth as a primary analytic focus. These factors may have shaped participants’ confidence, perceived safety, and uncertainty around physical activity, particularly as some participants described regular urate-lowering therapy as helping them feel more in control of gout and less concerned that activity would trigger a flare. Future research should examine how gout treatment and disease control influence physical activity engagement. Further research may also explore clinician perspectives to help identify barriers and opportunities for improving education and support. Future longitudinal research may clarify the relationship between physical activity and gout flares, including whether specific thresholds of activity or types of movement act as triggers.

In conclusion, this study challenges assumptions about inactivity, reveals gaps in clinical support, and underscores the need for more personalised guidance around physical activity in gout. These findings may inform future intervention development, and gout-specific physical activity guidance that supports people with gout to move safely, confidently, and meaningfully.

## Supplementary Information

Below is the link to the electronic supplementary material.

Supplementary Material 1

Supplementary Material 2

## Declarations

### Previous presentation

This work has not been previously submitted, presented or published as a congress abstract.

### Use of AI-assisted technology

Artificial intelligence-assisted technologies were not used in the drafting, editing, data collection, data analysis, interpretation of findings, or preparation of this manuscript.

### Editorial assistance

No external commercial editing support was used in the preparation or revision of this manuscript. .
