# Supplementary material for: Stigma, lost autonomy, reclaimed identity, and uncertainty around safe movement: a qualitative interview study of physical activity in gout
Source: Rheumatol Int. 2026 Jul 28;46(8):224. doi: 10.1007/s00296-026-06265-3 (PMC13415485; doi:10.1007/s00296-026-06265-3)
Supplement: Supplementary file 1 — Supplementary Material 1 [file 296_2026_6265_MOESM1_ESM.docx]

**SUPPLEMENTARY FILE 2**

**Interview guide**

Opening karakia [non-secular prayer/blessing] (as appropriate, to create a safe, focused space of respect and connection)

[***recording starts***]

**Background data**

- Before we start, I’m going to ask you a few questions about yourself and your gout.
- How old are you?
- What gender do you identify with?
- What is your ethnicity?
  - If Māori, what iwi or hapū do you identify with?
- How long ago were you told by a doctor that you had gout?
- How many gout attacks have you had in the last 6 months?
- Are you taking any medicines for your gout? What are they?
  - *Prompts: Do you take any pain relief for gout attacks and/or any ongoing maintenance therapy (i.e., urate lowering therapy))? If taking a urate lowering therapy: How long have you been taking this medication and how often do you take it? Do you manage or treat your gout in any other way (other than what the doctor prescribes)?*
- Have you got any other health conditions/health problems? What are they?

**Opening Questions: Experience of gout flares**

*[These opening questions were designed to get the participant thinking about their gout]*

We are going to start by talking about your experience with gout attacks.

- Can you tell me about your very first *[or most recent]* gout attack?
  - *Prompts: When did it happen? What was going through your mind at the time? What did it feel like? What did you do? Which joint(s) were involved? What were you doing at the time? How long did it last?*
- What happened after that?
  - *Prompts: Did you see a doctor and what did they do? What has your gout been like since your first attack?*

**PART A: Experience of physical activity**

We’re now going to talk about physical activity. For the purpose of this study, physical activity is any movement that uses your muscles and needs energy. So, this can include the obvious things like playing sports or going to the gym, but can also include household chores like cleaning, vacuuming, gardening or mowing the lawn. It can also include things like walking to the bus stop or walking to work. Or you might have a job that means you’re on your feet or moving about throughout the day.

- Can you tell me about your experience of physical activity?

*[Use of prompts was directed by the participants story-telling and emotional cues and used by the researchers as appropriate to elicit a richer story. Prompts may have included:]*

- - *If you think about everything that physical activity includes, what does your typical day look like when you’re not having a gout attack?*
  - *Do you ever set aside time in your day, or throughout your week that you dedicate to being physically active?*
  - *How important is being physical activity to you?*
  - *How does being physically active make you feel?*
  - *What is your favourite way to exercise or remain active?*
  - *How satisfied are you with your current physical activity level?*
  - *What do you enjoy most about being physically active?*

**PART B: Experience of gout flares and physical activity**

We’re now going to talk gout and physical activity.

- What’s the first thing that comes to mind when you think about gout and physical activity?

*[Use of prompts was directed by the participants story-telling and emotional cues and used by the researchers as appropriate to elicit a richer story. Prompts may have included:]*

- *If you think back to your last gout attack, how physically active were you during this time? Were you able to walk? Did you do any physical activity or exercise during your gout attack? What was going through your mind at the time? How did that make you feel?*
- *Thinking about all the times you’ve had a gout attack, has there been a particular attack that has affected your ability to be physically active the most? Which joint was involved? What was going through your mind at the time? How did it make you feel?*
- *How long after an attack ends, do you get back to your normal level of physical activity?*
- *In what ways do gout attacks make it more difficulty for you to exercise or be active? How does this make you feel?*
- *How has having gout held you back from doing certain activities? How does this make you feel?*
- *How has your level of physical activity changed compared to before you had gout? How does this make you feel?*
- *Has there ever been a time when you felt that physical activity or exercise has triggered a gout attack? Can you tell me about this attack? When did it happen? What did it feel like? Which joint(s) were involved? How long did it last? Can you describe the physical activity/exercise that you think triggered the attack? What was going through your find at the time?*
- *Do you ever worry that exercise will hurt your joints or give you a gout attack? Why do you think this? What activities do you avoid? How does this make you feel?*
- *Since your diagnosis of gout, have you ever been told by a doctor to change your physical activity levels or to be more active? Can you remember what advice they gave you? Why do you think they asked you to do this? How did this make you feel?*
- *How important do you think physical activity is for people with gout? Why do you think this?*

[*The interviewer then summarised the main points discussed in the interview and invited the participant to share anything further related to their experience of physical activity and gout*].

[***recording stops***]

Closing karakia [non-secular prayer/blessing] (as appropriate, to acknowledge contribution of the interviewer and interviewee, and bring a sense of completion)
